# Enhancement of HSA-pFSHβ production by disrupting *YPS1* and supplementing N-acetyl-L-cysteine in *Pichia pastoris*

**DOI:** 10.3389/fmicb.2022.998647

**Published:** 2022-12-21

**Authors:** Gang Luo, Zijian Geng, Buayisham Kuerban, Yingqing Xu, Jingjing Yang, Jiying Liu, Muwang Li

**Affiliations:** ^1^Jiangsu Key Laboratory of Sericultural Biology and Biotechnology, School of Biotechnology, Jiangsu University of Science and Technology, Zhenjiang, Jiangsu, China; ^2^Jiangsu Guiliu Animal Husbandry Group Co., Ltd., Xuzhou, Jiangsu, China

**Keywords:** *Pichia pastoris*, follicle-stimulating hormone, *YPS1*, NAC, *YPT7*

## Abstract

**Introduction:**

*Pichia pastoris* is widely used for the production of recombinant proteins, but the low production efficiency hinders its wide application in biopharmaceuticals. Moreover, many biopharmaceutical-like proteins are accompanied by degradation during secretory expression in *P. pastoris*.

**Objective:**

In this study, we used human serum albumin and porcine follicle-stimulating hormone β (HSA-pFSHβ) fusion protein as a model protein to investigate whether *YPS1* and *YPT7* gene disruption and *N*-acetyl-L-cysteine (NAC) supplementation have synergistic effects to inhibit the degradation of recombinant proteins.

**Results and discussion:**

Our results showed that *YPS1* gene disruption reduced the degradation of intact HSA-pFSHβ and increased the yield of intact protein in the culture medium and cells without affecting the integrity of the cell wall. Moreover, the beneficial effects of *YPS1* gene disruption were associated with the upregulation of the MAPK signaling pathway and maintenance of redox homeostasis. *YPS1* gene disruption and NAC supplementation had synergistic effects on HSA-pFSHβ production. In addition, disruption of vacuolar morphology by *YPT7* gene disruption or NH_4_Cl treatment affected the production of recombinant HSA-pFSHβ protein. Furthermore, *YPT7* gene disruption inhibited the processing of signal peptide in high-level produced HSA-pFSHβ strain. In conclusion, our results demonstrated that *YPS1* disruption could reduce the degradation of intact HSA-pFSHβ proteins, and synergistically increase the yield of intact HSA-pFSHβ with NAC supplementation. This study provided a valuable reference for reducing recombinant protein degradation and therefore improving the yield of recombinant proteins in *P. pastoris*.

## Highlights

- *YPS1* gene disruption could reduce the degradation of recombinant HSA-pFSHβ.
- Disrupting *YPS1* gene and NAC supplementation have a synergistic effect on HSA-pFSHβ yield.
- Vacuole affects the production of recombinant HSA-pFSHβ proteins.

## Introduction

The methylotrophic yeast *Pichia pastoris* is a realistic platform for the industrial production of recombinant proteins such as enzymes, growth factors, and antibodies for industrial and pharmaceutical use (Spohner et al., 2015; Arjmand et al., 2019; Matsuzaki et al., 2022). As a host, *P. pastoris* has the advantages of both eukaryotic and prokaryotic organisms, including high cell density fermentation and post-translational modifications (Liu et al., 2019; Karbalaei et al., 2020). Moreover, *P. pastoris* secretes low levels of endogenous proteins, a feature that facilitates the isolation and purification of recombinant proteins because they make up the majority of protein in the culture medium (Mattanovich et al., 2009). Unlike industrial enzymes, many proteins for biopharmaceutical use, such as Human interferon gamma, are not produced in high yield in *P. pastoris*, making it difficult to meet the demand for industrial production (Razaghi et al., 2017). Degradation of recombinant proteins, which cannot be effectively secreted into the culture medium, is a major reason for the low protein expression. (Sinha et al., 2005; Wu et al., 2013). Thus, reducing protein degradation is an effective approach to increase recombinant protein production in the culture medium.

Yapsin 1 (Yps1p), a member of the yapsin family, plays an important role in degrading recombinant protein in yeast (Kerry-Williams et al., 1998). Yps1p is a glycosylphosphatidylinositol (GPI)-anchored aspartic peptidases with an optimum pH of 4.0–5.0, and can recognize not only paired basic residues but also a monobasic residue accompanied by an additional basic amino acid at a specific upstream or downstream position (Yao et al., 2009). It has been reported that Yps1p has no apparent effect on cell phenotype and recombinant protein production, although it is important for the survival of thermal and cell wall stress in fungi (Guan et al., 2012; Sazonova et al., 2013; Wu et al., 2013). In *P. pastoris*, Yps1p is involved in the degradation of the proteolysis of recombinant proteins such as human interferon alpha 16 (Sazonova et al., 2013), and *YPS1* gene disruption improves the yield of HSA fusion protein in the culture medium (Yao et al., 2009). Thus, disruption of protein hydrolases is an effective means to inhibit the degradation of recombinant protein. Moreover, our previous study has shown that *N*-acetyl-L-cysteine (NAC) supplementation could improve extracellular human serum albumin and β subunit of porcine follicle-stimulating hormone fusion protein (HSA-pFSHβ) production and reduce intracellular HSA-pFSHβ degradation through increasing intracellular GSH content (Luo et al., 2021). Furthermore, the pro-secretory effect of NAC was also demonstrated on other recombinant proteins, such as HSA and interleukin 22 fusion protein (HSA-IL-22) and lipase. Whether *YPS1* gene disruption has a synergistic effect with NAC in enhancing recombinant protein production has not been reported.

In addition, as the endpoint for macromolecular degradation, vacuoles play a critical role in the degradation of recombinant protein (Li and Kane, 2009). Marsalek et al. (Marsalek et al., 2017) recently found that certain recombinant proteins are mistargeted to the vacuole for the degradation on their route to secretion. Disruption of vacuolar proteases such as proteinase A (Pep 4) is an effective way to reduce proteolytic degradation and thus improve secretion efficiency (Wu et al., 2013). Moreover, preventing recombinant proteins such as carboxylesterase (CES) or antibody fragment HyHEL-Fab from targeting vacuole by disrupting the vacuolar protein sorting component of homotypic fusion and protein sorting (HOPS) complex could improve its secretion (Marsalek et al., 2019). Ypt7 protein (Ypt7p) is a member of HOPS complex, which is localized to the vacuole for mediating the cargo arrival to the vacuole and plays an important role in degrading recombinant proteins expressed in *P. pastoris* (Haas et al., 1995). Given this background, we hypothesized that Yps1p and Ypt7p may have synergistic effect in reducing recombinant degradation.

Our previous study has shown that the yield of HSA-pFSHβ was significantly increased compared with pFSHβ in *P. pastoris* (Luo et al., 2018). However, a large amount of the recombinant protein cannot secreted into the culture medium and is degraded by hydrolases in the cells. In this study, we investigated the effect of *YPS1* and *YPT7* gene disruption and NAC supplementation on the degradation and production of recombinant proteins using HSA-pFSHβ as a model protein. Our results had shown that *YPS1* gene disruption has a synergistic effect with NAC supplementation to enhance recombinant HSA-pFSHβ protein production and reduce intact HSA-pFSHβ degradation. The beneficial effect of *YPS1* gene disruption on intact HSA-pFSHβ production and degradation was associated with upregulation of the MAPK signaling pathway and maintaining redox homeostasis. Moreover, Ypt7p was involved in signal peptide processing and the yield of HSA-pFSHβ was affected by vacuole morphology.

## Materials and methods

### Strains, plasmids and reagents

*Escherichia coli* strain Top10 (CWbio, Beijing, China), *P. pastoris* strain GS115-pPIC9K-HSA-pFSHβ (Termed F strain, HSA-pFSHβ gene ID: MH249035) constructed in our laboratory (Luo et al., 2018), and vectors pPICZA (Invitrogen, Shanghai, China) were used for cloning and heterologous expression. Plasmid BB3cN_pGAP_23*_pPFK300_Cas9 (Addgene #1000000136) was provided by Brigitte Gasser Lab of University of Natural Resources and Life Sciences Vienna (Gassler et al., 2019). *N*-acetyl-L-cysteine (NAC, Cat#A7250) were provided by Sigma-Aldrich. Restriction enzymes *Sal*I, *Xba*I and *Bgl*II were obtained from TAKARA Biotechnology Co., Ltd. (Dalian, China). Zeocin was purchased from Thermo Fisher Scientific (Waltham, MA, USA).

### Construction of expression plasmid of pPICZ-YPS1

pPICZ-YPS1 using for homologous recombination was constructed as below: An ∼0.5-kb DNA fragment containing N-terminal region of *YPS1* gene and an ∼0.6-kb DNA fragment containing C-terminal region were amplified from the genome of the GS115 strain using the primer pairs NY-*Xba*I-F + NY-*Sal*I-R and CY-*Bgl*II-F + CY-*Xba*I-R (all primers are shown in Table 1), respectively. The two fragments (*YPS1* N and *YPS1* C) were digested with *Xba*I/*Sal*I and *Bgl*II/*Xba*I, respectively. The digestion product ligated into the corresponding site of plasmid pPICZA to construct recombinant plasmid pPICZ-YPS1.

**TABLE 1:** Oligonucleotide primers used in this study.

Primers	Sequence (5′-3′)
NY-*Xba*I-F	GCATCTAGATTTCCGCTCAGCCAGATTTT
NY-*Sal*I-R	TAACGTCGACAACTAGTGCTAGTTCCAACG
CY-*Bgl*II-F	CGGAAGATCTCTCCTATGATTCGTCAAGAC
CY-*Xba*I-R	GCATCTAGAACTATACACACGCCGAGAAT
P1	GTTCCCGCGTGAAGAGAGATATA
P2	TTCCACTGTAGCACCCCCAAAAT
P3	TGCCACAGCCGTATCGGTAAGTT
P4	AGAGTTCCCTGGTCAGAACCCTT
A-sgRNA-struc-rev	CGCCATGCCGAAGCATGTTGCCCAGCCGGCGCCAGC GAGGAGGCTGGGACCATGCCGGCC
B-sgRNA-struc-rev	agaagacgcaagcaGTCCAAAGCTGTCCCATTCGCCATGCCGAAGCATGTTGCCCAGCCG
C-sgRNA-struc-rev	AGGCTGGGACCATGCCGGCCAAAAGCACCGACTCGG TGCCACTTTTTCAAGTTGATAACG
D-sgRNA-struc-fw	GTTTTAGAGCTAGAAATAGCAAGTTAAAATAAGGCTA GTCCGTTATCAACTTGAAAAAgT
1-Ypt 7-sgRNA-fw1	tgaagacgccatgGCTCCGCTGATGAGTCCGTGAGGACGAA ACGAGTAAGCTCGTCCGGA
2-Ypt 7-sgRNA-fw1	AAACGAGTAAGCTC CGGAGCAGACTTCCTAACAA GTTTTAGAGCTAGAAATAGCAAG
Ypt 7 - 300bp-F	TACTTCGAGGAATGAACAGC
Ypt 7 + 300bp-R	CAATGTTGACTGCTTCTTTG
DL-pFSHβ-F	CATCAACACTACTTGGTGTGCC
DL-pFSHβ-R	AGCACAACCTGGAACCTTAAC
DL-P.p-GAPDH-F	GGTATTAACGGTTTCGGACGTATTG
DL-P.p-GAPDH-R	GATGTTGACAGGGTCTCTCTCTTGG

### Verification of the *YPS1* gene disruption mutants

The plasmid of pPICZ-YPS1 was linearized with *Xba*I and transformed into *P. pastoris* strains GS115-pPIC9K-HSA-pFSHβ by electroporation with a Bio-Rad Gene Pulser Xcell (2 kV, 25 μF, 200 Ω) according to the modified electroporation method. The transformants were incubated in 1M sorbitol at 30°C for 1 h and then screened on YPDS plate (1% yeast extract, 2% peptone, 2% glucose and 1 M sorbitol) containing 100 μg/ml Zeocin for 2–4 days. *YPS1* disruptant on YPDS + Zeocin + plates were selected by PCR analysis with P1 + P2 and P3 + P4. The positive strains were named as Δyps1-5, Δ*yps1-39*, Δ*yps1-40* and Δ*yps1-41*.

### Construction of Δypt7 knock-out strains

Δ*ypt7* knockout strains were constructed by using CRISPR/Cas 9-based homology-directed genome editing as described by Gassler et al. (2019). In brief, a single guide RNA of *YPT7* gene was designed based on a protospacer adjacent motif (PAM) sequence identified in 50-200 bp upstream of the *YPT7* gene CDS on website.^1^ The ribozyme-sgRNA-fusion gene was generated by overlap extension PCR of six primers as showed in Table 1. For the CRISPR/Cas9-Δypt7 plasmid (BB3cN_pGAP_23*_pPFK300_Cas9 plasmid containing the sgRNA of *YPT7* gene), the Golden Gate assembly method was performed by using restriction enzyme *Bpi*I (Themo Fisher Scientific). The circular CRISPR/Cas9-Δ*ypt7* plasmid DNA was transformed in F strain or Δ*yps1* strains by electro-transformation following the *Pichia* expression system manual (Thermo Fisher Scientific) with minor modification. Briefly, we extended the ice bath time of circular CRISPR/Cas9-Δ*ypt7* plasmid and electro-competent cells mix to 20 min, and the incubation time to 3 h after transformation. The knockout (KO) strains were checked by direct PCR using primers YPT7-300bp-F and YPT7 + 300bp-R and sequencing. After confirmation of the *YPT7* gene deletion, true KO strains were passaged at least three times on YPD to lose the CRISPR/Cas9-Δ*ypt7* plasmid. The positive strains were named as F-Δ*ypt7*, Δ*yps1*-Δ*ypt7*.

### Recombinant protein production

F, Δ*yps1*, F-Δ*ypt7* and Δ*yps1*-Δ*ypt7* strains from YPD plates were selected to inoculate into 3 ml BMGY (1% yeast extract, 2% peptone, 1.34% YNB, 0.00004% biotin, 1% glycerol, 100 mM potassium phosphate, pH 6.0) at 30°C and shaken at 250 rpm. After 48 h, cells were harvested by centrifugation at 3,000 × *g* for 5 min, followed by decanting all the supernatant carefully and resuspending in 1 ml of BMMY (same as BMGY but replacing glycerol with 0.5% methanol) supplemented with or without 5 mM NAC to induce HSA-pFSHβ production for 72 h in shake flask. Methanol was added into the BMMY cultures every 24 h to maintain a concentration of 0.5%. There are three repeats per group. Every 12 h, OD_600_ was measured to test the growth rate of F, Δ*yps1*, F-Δ*ypt7* and Δ*yps1*-Δ*ypt7* strains. The recombinant HSA-pFSHβ protein in 5 μl culture medium was analyzed by using SDS-PAGE and Western blot. After 72 h induction, cells were collected for western blot analysis, chitin measurement and β-1,3-glucan measurement. For the ammonium chloride addition experiment, cells were dealt with 0.4 M NH_4_Cl for 72 h, and then the cells were collected for vacuolar staining.

### Extraction of intracellular proteins from recombinant *Pichia pastoris*

Intracellular proteins (including cytosolic proteins and membrane-associated proteins) were extracted using the method reported by Shen et al. (2012) with slight modification. Briefly, the cells were harvested by centrifugation at 12,000 × *g* for 3 min after inducing for 72 h and washed twice in phosphate buffered saline (PBS). Then 1 × 10^9^ cells were resuspended in 500 μl yeast breaking buffer (50 mM sodium phosphate, 2% protease inhibitor cocktail (Sigma P8215), 1 mM EDTA, and 5% glycerol (v/v)) and an equal volume of acid-washed glass beads were added. The cell walls were broken by vortexing 10 times for 1 min with 1 min resting in ice, followed by centrifugation at 16,000 × *g* for 20 min at 4°C. The supernatant containing cytosolic protein was collected for SDS-PAGE and Western blot analysis. The pellet was further resuspended with 200 μl yeast breaking buffer containing 2% (w/v) SDS and centrifuged at 4,000 × *g* for 5 min at 4°C, the supernatant containing membrane-associated protein was collected for SDS-PAGE and Western blot analysis.

### Polyacrylamide gel electrophoresis and Western blot analysis

The recombinant pFSH proteins in 5 μl culture medium or 1 × 10^9^ cells were analyzed on 12% SDS-PAGE and Western blot. Gels were stained by Coomassie blue R-250. For Western blot, a mouse anti-human FSHβ monoclonal antibody (Santa Cruz Biotechnology, Santa Cruz, CA, USA, 1:500) or mouse anti-His tag monoclonal antibody (Abclonal Technology, Beijing, China, 1:5,000) were used as the primary antibody to detect HSA-pFSHβ. For phosphor-Mpk1 and Mpk1, blots were probed with phospho-p44/42 MAPK (Erk1/2) (Cell Signaling Technology cat#4370, Shanghai, China, 1:2,000) and Mpk1 antibody (D-1) (Santa Cruz Biotechnology, Inc., Dallas, TX, USA), respectively. The secondary antibody (Zhongshan jinqiao Biotech, Beijing, China, diluted 1:5,000-1:10,000) was horseradish peroxidase (HRP) conjugated goat anti-mouse IgG and anti-rabbit IgG. The immunoreactive proteins on the blots were visualized with ECL and imaged on an Image Quant LAS 4000 instrument. Western analysis data are representative of triplicate experiments. Densitometric analysis of protein band on SDS-PAGE and Western blot was performed with ImageJ.^2^ The values of the protein band were normalized to the intact HSA-pFSHβ protein band in the culture medium of F strain. For Mpk1 detection, the optical density of pMpk1 (p42/44) and Mpk1 protein band were normalized to Mpk1 and GAPDH, respectively, and *t*-test was used to measure statistical significance.

### Phenotypic analysis

The phenotypic analysis of GS115, F strain and Δ*yps1* strains were performed following the method reported by Guan et al. (2012). Briefly, cells were grown overnight in liquid YPD (1% (w/v) yeast extract; 2% (w/v) peptone, and 2% (w/v) glucose) medium at 30°C. Then equal amounts of cells (approximately 5 × 10^7^ cells) were diluted in a series of 10-fold magnitude (from 10^–1^ to 10^–5^ relative to the initial culture) in water. Aliquots (2 μl) of 10-fold serial dilutions were spotted on the YPD plates containing 30 μg/ml Calcofluor White (CFW) or Congo red (CR). The plates were incubated at 30°C for 2–4 days and photographed. There were three repeats for each strain.

### Chitin staining and measurement

The cell wall chitin contents were measured following the method reported by Sun et al. (2014). Briefly, cells cultured with YPD for 24 h were collected by centrifugation at 3,000 × *g* for 5 min, and then stained for 5 min in 1 ml PBS buffer containing 20 μg/ml Calcofluor White (CFW). The CFW-stained cells were washed with PBS buffer and resuspended in 1 ml fresh PBS buffer. The fluorescence density (FLU) of the cells was determined using SpectraMax i3 fluorescence microplate reader (excitation wavelength 325 nm, emission wavelength 435 nm). The relative fluorescence density was expressed as the FLU divided by the number of examined cells, which were measured using a microplate reader.

### Quantitative β-1,3-glucan measurement

The relative amount of β-1,3-glucan was measured using aniline blue as described previously by Guan et al. (2012). In brief, cells were grown in YPD medium to OD_600_ 1.0 and 5.0 × 10^7^ cells were harvested by concentration at 6,000 × g for 3 min. Then washed the cells twice with 500 μl TE (10 mM Tris–HCl, 1 mM EDTA, pH 8.0) and resuspended them in 250 μl of TE. 50 μl NaOH (6 mol/L) was added to the cells and gently mix the tube. The cells were incubated at 80°C for 30 min followed by the addition of 1.05 ml of AB mix (0.03% aniline blue (Shanghai Sangon), 0.49 mol/L glycine and 0.18 mol/L HCl, adjusting pH 9.5 by NaOH). Then incubated the tube at 50°C for 30 min. The fluorescence of β-1,3-glucan was quantified using a SpectraMax i3 fluorescence microplate reader with an excitation wavelength at 386 nm, and an emission wavelength at 460 nm.

### Fluorescence microscopy

The vacuolar membranes of F and KO strains (including Δ*yps1-5*, Δ*yps1-39*, Δ*yps1-40*, *F*-Δ*ypt7* and Δ*yps1*-Δ*ypt7*) were stained by FM4-64 (Thermo Fisher Scientific) using the method reported by Delic et al. (2012) with slight modification. In brief, the strains were inoculated in YPD medium and grown to OD_600_ to 0.8–1.6. The cells were harvested at 700 × g for 3 min, and resuspended in 200 μl YPD containing 24 μM FM4-64 solution (diluted from a 16 mM stock solution in DMSO). After 15 min incubation at 30°C shaking at 170 rpm, the solution was replaced by a fresh YPD medium, and the cells were incubated for 30–60 min with shaking at 30°C. Finally, the cells were washed with YPD and observed under a fluorescence microscope using a × 100 oil immersion objective with exciter filter BP530-550 and barrier filter BA575-IF (Olympus IX3 equipped with U-FGW filter set).

### Intracellular reactive oxygen species level

The intracellular ROS levels in F and Δ*yps1* strains were measured by using a Reactive Oxygen Species Assay Kit (Beyotime Biotechnology, China). Briefly, the cells were harvested by centrifugation at 700 × *g* for 3 min after inducing for 72 h and washed twice in PBS. OD_600_ was measured and 5 × 10^7^ cells were incubated with DCFH-DA for 30 min at 30°C with 250 rpm shaking and then measured at 488 nm excitation wavelength and 525 nm emission wavelength by a fluorescence spectrophotometer (SpectraMax i3). The experiment was performed in three biological replicates.

### Determination of gene copy numbers

The genome of F and Δ*yps1* strains was extracted using method according to the user manual of Invitrogen’s Multi-Copy *Pichia* expression kit. The Quantitative real-time PCR was used to determined HSA-pFSHβ gene copy numbers by using the CFX96 real-time system (Bio-Rad, Beijing, China) in a reaction mixture containing (in a total volume 10 μl): 2 × Taq Pro Universal SYBR qPCR Master Mix (Vazyme, Nanjing, China), forward and reverse primers, cDNA and ddH_2_O. PCR was performed at 95°C for 30 s, followed by 39 cycles of 95°C for 10 s and 60°C for 30 s, and a melting curve was constructed at the end of the amplification. Triplicate samples of each template were analyzed. The expression levels of HSA-pFSHβ in F and Δ*yps1* strains were determined using qPCR analysis with primers DL-pFSHβ-F + DL-pFSHβ-R and primers DL-P.p-GAPDH-F + DL-P.p-GAPDH-R were used for the reference gene.

## Results

### Disruption of *YPS1* gene by homologous recombination

**FIGURE 1:**
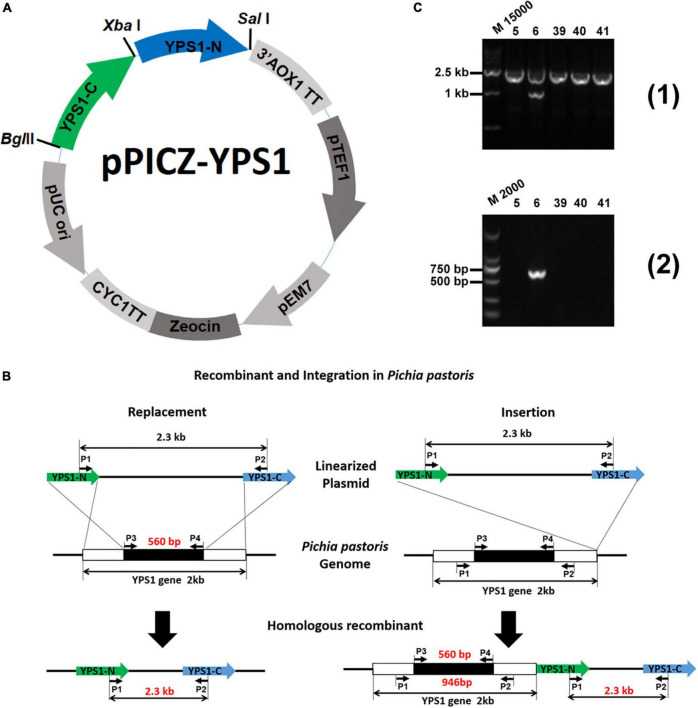
Schematic representation of experimental procedures for *YPS1* gene disruption and identification (Luo, 2019). **(A)** The map of the pPICZ-YPS1 plasmid was constructed in this study. **(B)** Homologous recombination and integration process of linearized pPICZ-YPS1. **(C)** The PCR products were amplified from the genome of transformants using the primer pairs P1 + P2 (1) and P3 + P4 (2). Lane M15000: DL15000 DNA Marker, lane M2000: DL2000 DNA Marker, lane 5, 6, 39, 40 and 41: Zeocin + transformants.

The vector of pPICZ-YPS1 used for *YPS1* mutation was constructed by replacing the AOX1 promotor in the pPICZA plasmid with *YPS1* gene homology arms as shown in Figure 1A. A ∼3.4 kb linearized DNA fragment was obtained following the digestion with enzyme *Xba*I. Replacement or insertion events would occur when high-level produced HSA-pFSHβ strain (named F strain) was transformed with the linearized DNA fragment that containing N-terminal and C-terminal homology arms of *YPS1* gene (Cregg et al., 1985, 1989). *YPS1* gene is disrupted when the replacement event occurred in yeast transformants, and only a 2.3 kb fragment is detected by PCR using primers P1 + P2 (Figure 1B). However, an additional 946 and 560 bp fragments are detected by primers P1 + P2 and P3 + P4 (all primers used in this study are shown in Table 1), respectively, when the insertion event occurred (Figure 1C). Four positive mutants named Δ*yps1-5*, Δ*yps1-39*, Δ*yps1-40* and Δ*yps1-41* were obtained successfully from 100 monoclonal yeasts on YPDS plates.

### Cell growth and cell wall composition of Δ*yps1* strains

**FIGURE 2:**
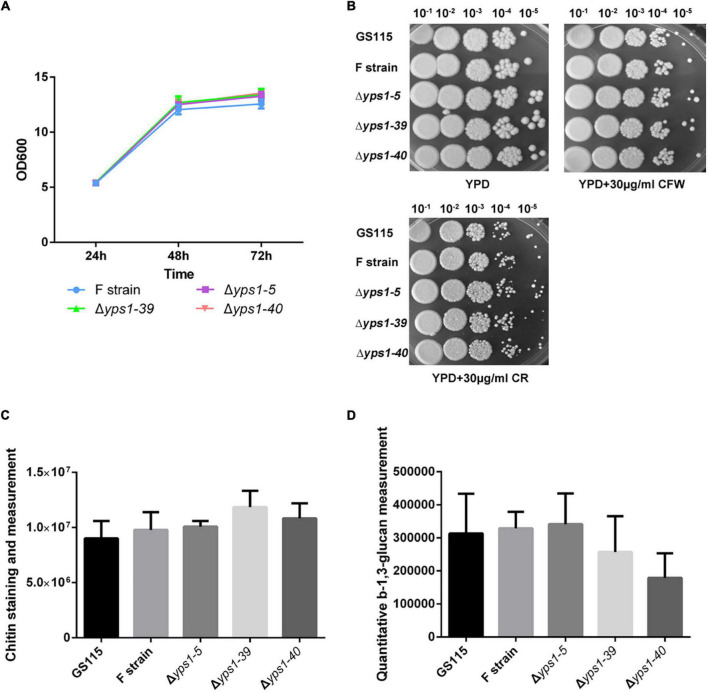
Cell growth and phenotypic analysis of *yps1*-disrupted strain. **(A)** Cell growth characteristics of *yps1*-disrupted strain versus wild-type strain in the BMMY culture medium. **(B)** Sensitivity of *yps1*-disrupted cells to CR and CFW. GS115, F, and *yps1*-disrupted strains were grown to mid-log phase and normalized to an OD_600_ of 1.0. The cells were diluted five times in a 10-fold series, and 2 μl of each dilution were spotted on YPD plates containing 30 μg/ml of CR or CFW. **(C)** Chitin staining and measurement. The cell grown in YPD at 24 h were collected by centrifugation at 3,000 × *g* for 5 min, and cells were stained with 20 μg/ml CFW. **(D)** Relative content of β-1,3-glucan. Cells were grown in YPD medium to OD_600_ 1.0 and 5.0 × 10^7^ cells were harvested by concentration at 6,000 × g for 3 min. The β-1,3-glucan content in the cell wall was measured with aniline blue. Three biological replicates were performed because of the low precision of the β-1,3-glucan measurement with aniline blue. The contents of chitin and β-1,3-glucan are no statistical difference between wild-type and *yps1*-disrupted strain. Error bars indicate means ± standard deviation (SD) (*n* = 3).

To investigate whether the disruption of *YPS1* gene has any effect on cell wall integrity, we tested the cell growth rate and cell wall composition of Δ*yps1* strains (including Δ*yps1-5*, Δ*yps1-39* and Δ*yps1-40* strains) versus the wild-type strain (F strain) in shake-flask level. Compared with F strain, the biomass production of cells indicated by OD_600_ of three mutant strains did not significantly change in the BMMY culture medium (Figure 2A). Moreover, both Calcofluor white (CFW) and Congo red (CR) reagents do not interfere with the cell growth of Δ*yps1* strains, compared with the parental F strain or GS115 in YPD plates with 30 μg/ml CFW or CR (Figure 2B). Moreover, the chitin and β-1,3-glucan contents in the cell wall of Δ*yps1* strains did not change significantly compared to F strain (Figures 2C, D), indicating that *YPS1* gene disruption does not affect the cell wall composition in *P. pastoris*. Those results indicated that the *YPS1* gene is not essential for *P. pastoris* survival during the induced expression phase of HSA-pFSHβ.

### Effect of *YPS1* gene disruption on the degradation and production of hSA-pFSHβ in shake flask cultivation

**FIGURE 3:**
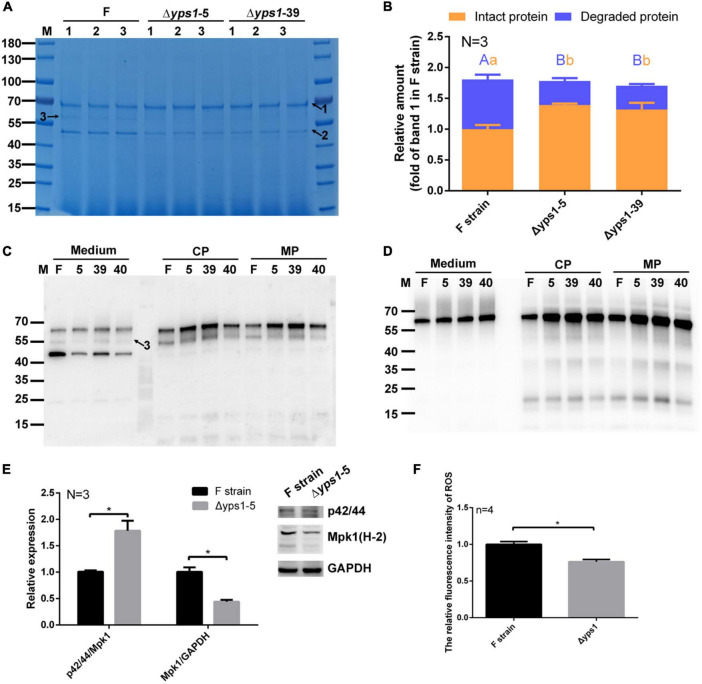
SDS-PAGE and Western blot analysis of HSA-pFSHβ protein in culture medium or cell of Δ*yps1* strains. **(A)** SDS-PAGE analysis of recombinant HSA-pFSHβ protein in the culture medium. F, Δ*yps1-5* and Δ*yps1-39* represent the culture medium of F, Δ*yps1-5* and Δ*yps1-39* strains induced for 72 h. Numbers 1, 2 and 3 below the horizontal line represent three replicate samples of the same strain. Arrow 1, HSA-pFSHβ protein; arrow 2, HSA truncated fragment; arrow 3, degraded fragment of HSA-pFSHβ. **(B)** The relative amounts of recombinant protein in figure. The values were normalized to the intact HSA-pFSHβ protein band (indicated by arrow 1) in the culture medium of F strain. Three repeats were used for data quantification. The same letters (a, b for intact protein and A, B for degraded protein) indicate no significant difference between the wild-type strain and *YPS1*-disrupted strain, and different letters indicate statistically significant differences (*p* < 0.05). **(C,D)** Western blot analysis of HSA-pFSHβ protein in the culture medium or cells (including cytosolic and membrane-associated proteins) reacted with mouse 6 × His tag monoclonal antibody **(C)** and human FSHβ monoclonal antibody **(D)**. CP, cytosolic protein; MP, membrane-associated protein. **(E)** Blots were probed with antibodies specific for phosphorylated forms of Mpk1 and Mpk1. The data shown are representative of three independent experiments. The results presented in the histogram reflect the fold-change in Mpk1 phosphorylation and Mpk1 in *YPS1* mutant compared with that of the wild-type cells, based on band density in arbitrary units as quantified by densitometry analysis. **(F)** ROS level within F and Δ*yps1* strains after 72 h induction. *p* values were calculated using Student’s *t*-test with *p* < 0.05 considered statistically significant (marked with *). Error bars represent means ± standard deviation (SD) (*n* = 3).

To investigate whether *YPS1* gene disruption reduces the degradation of intact HSA-pFSHβ (indicated by arrow 1 in Figure 3A) in the culture medium, HSA-pFSHβ fusion protein (Connected via flexible linker GGGGS) was expressed in culture medium of F strain and Δ*yps1* strains. As shown in Figure 3A, two protein bands were detected in the culture medium. The protein band of approximately 68 kDa is an intact HSA-pFSHβ fusion protein, and the protein band of approximately 45 kDa is a well-known HSA degraded fragment (indicated by arrow 2), which had been confirmed by mass spectrometry analysis (Luo et al., 2018). Compared with F strain, the yields of intact HSA-pFSHβ protein in the culture medium of Δ*yps1* strains were significantly improved (1.39 ± 0.02 and 1.32 ± 0.11 versus 1.00 ± 0.07; Figure 3B and Supplementary Figure 1), and the yields of degraded fragment (arrow 2 on Figure 3A) were significantly decreased (0.39 ± 0.05 and 0.38 ± 0.03 versus 0.81 ± 0.08). In addition, the degraded fragment indicated by arrow 3 (approximately 58 kDa) in Figure 3A was significantly reduced or even disappeared in the culture medium of Δ*yps1* strains, compared with F strain. Those results indicated that the degradation of HSA-pFSHβ fusion protein could be reduced by disrupting the *YPS1* gene.

We detected the yield of recombinant protein in cells (including cytosolic proteins and membrane-associated proteins) by Western blot, using antibodies specifically reacted with his-tag or FSH. As shown in Figures 3C, D, the protein band approximated 55 kDa (indicated by arrow 3), which could be detected by His-tag antibody rather than human FSHβ antibody, is a HSA-pFSHβ degraded fragment. This fragment was degraded from the C-terminal, which could be inferred from the 6 × his sequence attached to the N-terminal of HSA-pFSHβ (Luo et al., 2018). Unlike in the culture medium, almost no ∼45 kDa degraded fragment was detected by his-tag in the cells. Compared with that in the F strain, the intact HSA-pFSHβ fusion protein in cells of Δ*yps1* strains is increased obviously (Figure 3C), indicating that a portion of the HSA-pFSHβ fusion protein was cleaved intracellularly during the secretory process.

### MAPK signaling pathway is involved in the inhibition of *YPS1* gene disruption on hSA-pFSHβ degradation

MAPK signaling pathway is mutually regulated with the *YPS1* gene and plays an important role in the cell wall integrity pathway (Chow et al., 2018). To explore whether the inhibition of disrupting the *YPS1* gene on HSA-pFSHβ degradation was related to MAPK pathway, the expression and phosphorylation level of Mpk1 were detected in F and Δ*yps1* strains. As shown in Figure 3E, compared with F strain, the expression level of Mpk1 (Mpk1/GAPDH) (0.44 ± 0.04 versus 1.01 ± 0.08) is significantly decreased and the phosphorylation of Mpk1 (p42/44/Mpk1) (1.78 ± 0.19 versus 1.01 ± 0.02) was significantly improved in the cell of Δ*yps1* strains (*p* < 0.05). This result implicated that the beneficial effect of *YPS1* gene disruption on HSA-pFSHβ degradation is related to the upregulation of the MAPK slt2/Mpk1 signaling pathway, consistent with that in *S. cerevisiae* (Gagnon-Arsenault et al., 2006). It has been reported that methanol induction enhanced the upregulation of the MAPK signaling pathway in *P. pastoris*, which may regulate the alcohol oxidase1 (AOX1) promoter via regulatory factors activated by methanol-mediated stimulation (Zhang et al., 2020). In our study, the AOX activity did not improve with the upregulation of the MAPK pathway in the cell of Δ*yps1* strains versus F strain (Supplementary Figure 2). In short, the MAPK slt2/Mpk1 signaling pathway is involved in the alleviated effect of *YPS1* gene disruption on the degradation of HSA-pFSHβ.

It has been reported that the robust mutant of lipase not only upregulated MAPK signaling pathway but also reduced intracellular reactive oxygen species (ROS) levels and enhanced oxidative stress tolerance of *P. pastoris* (Lin et al., 2021). To further investigate the mechanism of disrupting *YPS1* gene to inhibit HSA-pFSHβ degradation, we examined the ROS levels within F and Δ*yps1* strains after 72 h of induction. As shown in Figure 3F, the intracellular ROS levels were significantly reduced in Δ*yps1* strain, compared with that in F strain. This result indicated that oxidative stress caused by H_2_O_2_, a byproduct of methanol metabolism, could be alleviated by disruption of *YPS1* gene.

### Effect of *N*-acetyl-L-cysteine on the production and secretion of intact hSA-pFSHβ in Δ*yps1* strains

**FIGURE 4:**
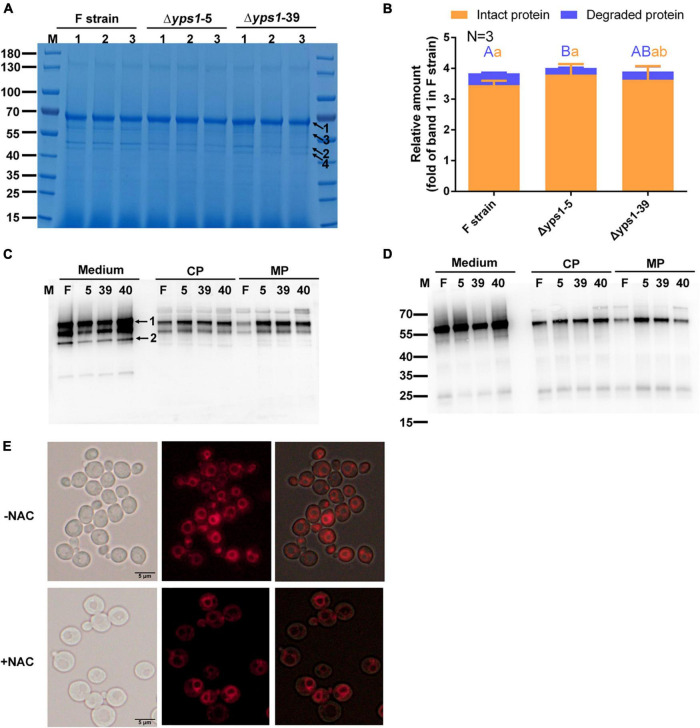
Effect of *N*-acetyl-L-cysteine (NAC) on the secretion of intact HSA-pFSHβ in Δ*yps1* strains. **(A)** SDS-PAGE analysis of recombinant HSA-pFSHβ protein in culture medium treated with 5 mM NAC for 72 h. Numbers 1, 2 and 3 below the horizontal line represent three replicate samples of the same strain. Arrow 1, HSA-pFSHβ protein; arrow 2, HSA truncated fragment; arrows 3 and 4, degraded fragments of HSA-pFSHβ. **(B)** The relative amounts of protein bands of figure were estimated using Image J analysis software. The values were normalized to the intact HSA-pFSHβ protein band (indicated by arrow 1 in Figure 3A) in the culture medium of the F strain. Error bars represent means ± standard deviation (SD) (*n* = 3). **(C,D)** Western blot analysis of HSA-pFSHβ protein in the culture medium or cells treated with NAC. Blots were probed with antibodies specific reacted with mouse 6 × His tag monoclonal antibody **(C)** and human FSHβ monoclonal antibody **(D)**. **(E)** Vacuolar morphology of F strain supplemented with or without 5 mM NAC.

To promote the secretion of intracellular increased intact HSA-pFSHβ fusion protein, we detected the production of HSA-pFSHβ in the culture medium or cell of Δ*yps1* strains supplementing with 5 mM NAC. As shown in Figure 4A, another protein band (indicated by arrow 4) was detected on SDS-PAGE gel, and a similar faint protein band can be seen in Figure 3A, indicating that the band became more pronounced after NAC supplementation. Compared with that of F strain, the yield of intact HSA-pFSHβ protein in the culture medium of Δ*yps1* strains is improved by approximately 5–10% by NAC supplementation (3.80 ± 0.34 and 3.63 ± 0.44 versus 3.46 ± 0.14; Figure 4B and Supplementary Figure 1), based on densitometric analysis of the protein bands on SDS-PAGE in Figure 4A (the values were normalized to the protein band indicated by arrow 1 in Figure 3A). Moreover, the 45 kDa degraded fragment (arrow 2 in Figure 4A) decreased by approximately 30–40% in the culture medium of Δ*yps1* strains, versus F strain (0.22 ± 0.01 and 0.27 ± 0.17 versus 0.38 ± 0.02). However, the intracellular intact HSA-pFSHβ protein bands in Δ*yps1* strains are still brighter than that in F strain (Figures 4C, D), indicating that NAC did not fully promote the secretion of intracellular increased intact HSA-pFSHβ protein.

Compared with the protein band in Figure 3B, the intact HSA-pFSHβ protein is improved 2.5-fold in the culture medium of F strain and 1.7-fold in the culture medium of Δ*yps1* strains, and the 45kDa-degraded fragment is decreased by 1.1-fold in the culture medium of F strain, versus 0.6-fold in that of Δ*yps1* strains (Figure 4B). These results indicated that NAC reduces the amplitude of increase in intact protein or decrease of degraded fragments caused by *YPS1* gene disruption. Thus, NAC could improve the production of intact HSA-pFSHβ protein in the culture medium, reduce the proportion of degraded fragments, and partially override the inhibited effect of *YPS1* gene disruption on intact HSA-pFSHβ degradation. Compared to NAC-treated F strain or Δ*yps1* strains without NAC treatment, the yield of intact HSA-pFSHβ protein is elevated and degraded fragments production are decreased in the culture medium of Δ*yps1* strains supplemented with NAC, suggesting that NAC and *YPS1* gene disruption have a synergistic effect on HSA-pFSHβ production. Interestingly, we found that the proportion of vacuole to whole cell is significantly reduced (Figure 4E).

### Effect of *YPT7* gene disruption on the degradation of hSA-pFSHβ

**FIGURE 5:**
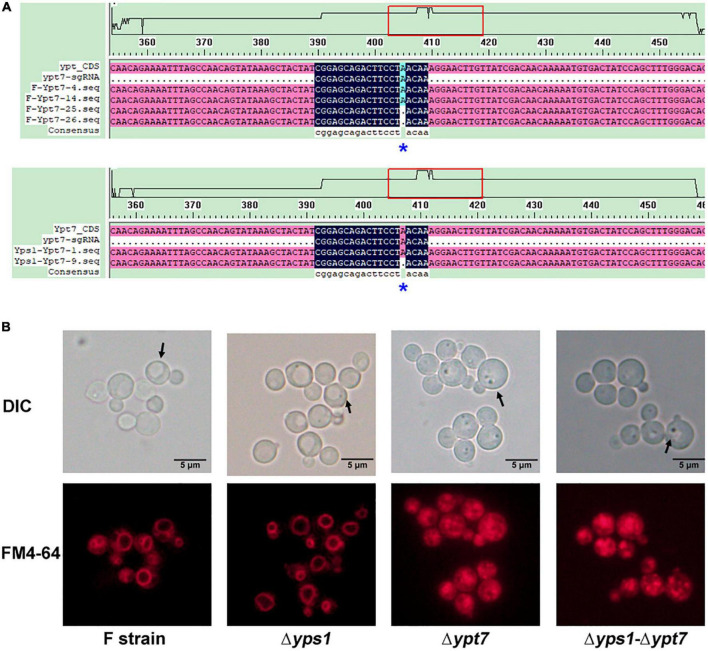
Identification and phenotype of *YPT7*-disrupted strains. **(A)** Knock out the *YPT7* gene by CRISPR-Cas 9 in F (Upper) and Δ*yps1* (Lower) strains. **(B)** Vacuolar morphology in F and mutant strains. Upper, Nomarski optics; lower, FM4-64 fluorescence.

To further inhibit the degradation of intracellular HSA-pFSHβ, we have disrupted the *YPT7* gene by CRISPR-Cas 9 as reported by Gassler et al. (2019). As shown in Figure 5A, an adenine was deleted at 39-base-pair of the *YPT7* gene, resulting in frameshift mutation of the *YPT7* gene in F and Δ*yps1* strains, named F-Δ*ypt7* and Δ*yps1*-Δ*ypt7*, respectively. To further confirm to the positive yeast clones, the vacuolar morphology of F-Δ*ypt7* and Δ*yps1*-Δ*ypt7* strains was checked by FM4-64 staining (*N*-(3-triethylammoniumpropyl)-4-(p-diethylaminophenylhexatrienyl)-pyridinium 2Br). As shown in Figure 5B, the size of the vacuole is significantly reduced in F-Δ*ypt7* and Δ*yps1*-Δ*ypt7* strains, compared with F or Δ*yps1* strains. Moreover, there are many multiple small vacuole-like compartments in cells of F-Δ*ypt7* and Δ*yps1*-Δ*ypt7* strains. This result is consistent with Marsalek’s report, suggesting that vacuolar morphology can be used as a marker for *YPT7* gene disruption (Marsalek et al., 2019). It has been reported that the small compartments were the accumulation of intracellular vesicles delivered via endocytosis or vacuolar targeting pathways, which fail to fuse with vacuole (Marsalek et al., 2019).

**FIGURE 6:**
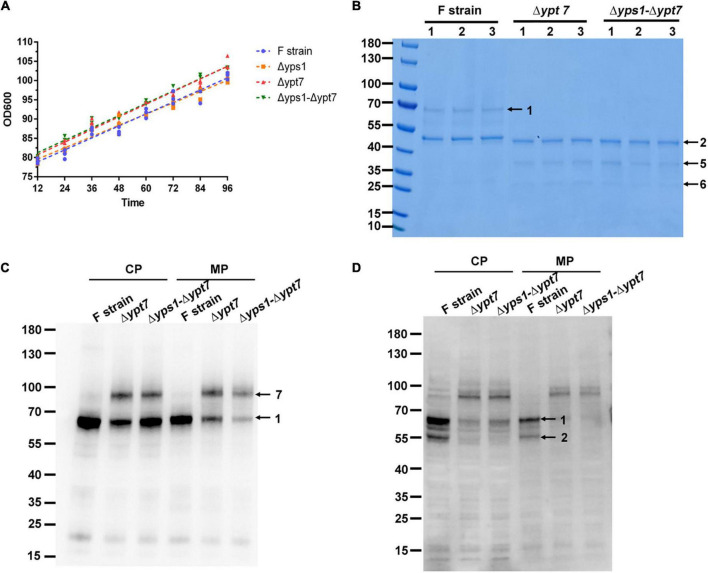
Cell growth and HSA-pFSHβ production in Δ*ypt7* and Δ*yps1*-Δ*ypt7* strains. **(A)** The cell growth curve (OD_600_) of Δ*yps1* and Δ*yps1*-Δ*ypt7* strains. The same amount of cells were inoculated into the induction medium. A linear regression of OD_600_ values against time was performed. Results are means ± standard deviations (*n* = 3). **(B)** Effect of *YPT7* gene disruption on the yield of HSA-pFSHβ in the culture medium of F, Δ*ypt7*, and Δ*yps1*-Δ*ypt7* strains. Arrow 1, HSA-pFSHβ protein; arrow 2, HSA truncated fragment; arrows 5 and 6, degraded fragments of HSA-pFSHβ. **(C,D)** Western blot analysis of HSA-pFSHβ protein in cells of *YPT7* gene disruption strains. Blots were probed with antibodies specific for mouse 6 × His tag monoclonal antibody **(C)** and human FSHβ monoclonal antibody **(D)**. Arrow 7, not fully processed HSA-pFSHβ containing the full-length α-factor signal peptide sequence. CP, cytosolic protein; MP, membrane-associated protein.

After confirming positive yeast clones, the recombinant HSA-pFSHβ protein of the *YPT7* disrupted strain was expressed at the shake flask level. The same amount of cells were inoculated into the BMMY culture medium. The cell densities of OD_600_ was measured every 12 h and the growth curve of F-Δ*ypt7* and Δ*yps1*-Δ*ypt7* were analyzed by linear regression. As shown in Figure 6A and Supplementary Figure 3, *YPT7* gene disruption does not affect the growth rate of cells dealt with or without NAC, which was confirmed by the similar slopes of F, Δ*yps1*, F-Δ*ypt7* and Δ*yps1*-Δ*ypt7* strains. The biomass production of cells in F-Δ*ypt7* and Δ*yps1*-Δ*ypt7* strains were significantly increased at different time, compared with that in F strain and Δ*yps1* strains, respectively (Supplementary Table 1). In total, these results indicate that *YPT7* gene disruption does not affect cell growth, which was consistent with the result in *S. cerevisiae* that *YPT7* gene disruption did not impair cellular growth at different temperature (Wichmann et al., 1992).

It has been reported that *YPT7* gene disruption could improve the secretion of HyHEL-Fab antibody and carboxylesterase (Marsalek et al., 2019). To investigate whether *YPT7* gene mutation has a similar effect on the secretion of HSA-pFSHβ, we detected the yield of recombinant protein in culture medium and cells. As shown in Figure 6B, no intact HSA-pFSHβ band (arrow 1) could be detected in the culture medium of F-Δ*ypt7* and Δ*yps1*-Δ*ypt7* strains. In addition, another two protein bands (arrows 5 and 6) appears when the *YPT7* gene was disrupted. To explore the disappearance of intact HSA-pFSHβ in the culture medium, we detected the intracellular protein with FSHβ and His-tag antibodies. The density of intracellular intact HSA-pFSHβ protein band is obviously reduced in cells of F-Δ*ypt7* and Δ*yps1*-Δ*ypt7* strains, compared with that of the wild-type strain (arrow 1 on Figure 6C). Those results indicated that *YPT7* gene disruption affects the production of intact HSA-pFSHβ protein. Interestingly, another protein band between 70 and 100 kDa (indicated by arrow 7) was recognized by FSHβ antibody in cells of F-Δ*ypt7* and Δ*yps1*-Δ*ypt7* strains (Figure 6C). Based on the size of molecular weight, we speculated that the protein band (indicated by arrow 7) may represent not fully processed HSA-pFSHβ containing the full-length α-factor signal peptide sequence (molecular weight approximately ∼18.3–21.3 kDa). In addition, we found that the ∼45 kDa degraded fragment is significantly decreased in the cells of F-Δ*ypt7* and Δ*yps1*-Δ*ypt7* strains, compared with that of F strain (Figure 6D), suggesting that Ypt7p participates in the degradation of HSA-pFSHβ. In short, we found that *YPT7* gene disruption during the production of HSA-pFSHβ could inhibit the process of α-factor signal peptide sequence.

**FIGURE 7:**
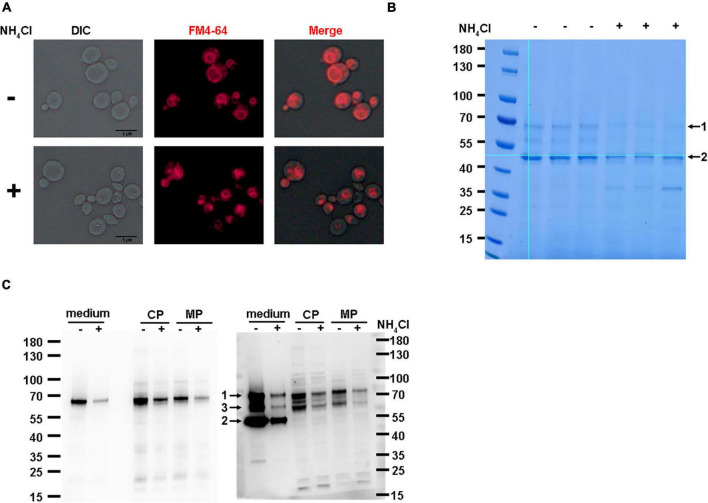
Effect of NH_4_Cl supplementation on the production of HSA-pFSHβ. **(A)** Effect of NH_4_Cl supplementation on vacuolar morphology in F strain. **(B)** SDS-PAGE analysis of HSA-pFSHβ protein in cells of F strain. Arrow 1 and 2 represent the intact HSA-pFSHβ protein and HSA-truncated degradation fragment, respectively. **(C)** Western blot analysis of HSA-pFSHβ protein in cells of F strain. Blots were probed with antibodies specific for mouse 6 × His tag monoclonal antibody (left) and human FSHβ monoclonal antibody (right). CP, cytosolic protein; MP, membrane-associated protein. +, represent culture medium supplementing with 0.4 M NH_4_Cl, otherwise marked -. Arrow 3, degraded fragment of HSA-pFSHβ.

Chemicals such as ammonium chloride (NH_4_Cl) have a similar effect on vacuolar morphology. To investigate whether vacuole morphology is the main reason for the inhibition of recombinant HSA-pFSHβ production by *YPT7* gene disruption, we tested the effect of NH_4_Cl on HSA-pFSHβ production. As shown in Figure 7A, 0.4 M NH_4_Cl supplementation could reduce vacuolar sizes of F strain during methanol induction. Moreover, the yield of intact HSA-pFSHβ protein and the main degradation fragment in the culture medium and cells are obviously reduced by supplementing NH_4_Cl (Figures 7B, C), suggesting that reducing vacuolar size by NH_4_Cl supplementation affects the production of recombinant HSA-pFSHβ protein. In addition, the protein band 7 in Figure 6C could not be detected in Figure 7C, indicating that NH_4_Cl did not affect the processing of the α factor signal peptide. In conclusion, these results suggest that the reduction of vacuolar size is one of the main reasons why *YPT7* gene disruption reduces extracellular HSA-pFSHβ protein production (Figure 7C).

## Discussion

Many recombinant proteins produced by *P. pastoris*, especially recombinant enzymes, have been widely used in food and feed applications (Spohner et al., 2015). However, most of the approved drugs are produced by Chinese hamster oocyte (CHO), *E. coli* and *Saccharomyces cerevisiae*, and few drug proteins are expressed by *P. pastoris* (Walsh, 2014). The low-level production of recombinant proteins is a main limiting factor preventing their widespread use in biopharmaceuticals (Razaghi et al., 2017). Our previous studies have shown that the yield of recombinant proteins was increased by fusing with truncated human serum albumin protein, but the degraded phenomenon was accompanied by an increase in the yield of recombinant proteins (Luo et al., 2018). Similar degradation has been observed in the production of other recombinant proteins (Wu et al., 2013). In this study, we used HSA-pFSHβ as a model protein to investigate whether *YPS1* and *YPT7* gene disruption and NAC supplementation have synergistic effects to inhibit the degradation of recombinant proteins.

Both Calcofluor white (CFW) and Congo red (CR) was thought to interfere with cell wall assembly by competitively binding chitin in the cell wall of yeast (Ram and Klis, 2006). In *S. cerevisiae*, the *YPS1* gene mutant is hypersensitive to CR, a dye that interferes with both β-1,3-glucan and chitin fibril formation in the cell wall (Kopecka and Gabriel, 1992; Krysan et al., 2005). Moreover, as cell wall-perturbing dyes, CFW and CR improved the expression of *YPS1* gene (Krysan et al., 2005). In contrast to *S. cerevisiae*, *YPS1* gene mutants of *P. pastoris* grew normally in the presence of CFW or CR (Guan et al., 2012; Naranjo et al., 2019). Our results had shown that the growth phenotype of Δ*yps1* strains is not affected in the presence of two cell wall-disrupting reagents (Kang et al., 1998; Yao et al., 2009).

In *Saccharomyces cerevisiae*, Yps1p could cleave more than 90% recombinant protein in the culture medium, and disruption of the *YPS1* gene or supplementation of an aspartic protease inhibitor could inhibit the degradation of recombinant protein (Kang et al., 1998). Moreover, the degradation of secreted HSA fusion protein could also be reduced by disrupting the *YPS1* gene in *P. pastoris* (Yao et al., 2009; Wu et al., 2013). Our results indicated that Yps1p partially degrades recombinant HSA-pFSHβ fusion protein in cell and culture medium, and *YPS1* gene disruption could reduce HSA-pFSHβ degradation. Moreover, the effect of *YPS1* gene disruption on increasing the production of HSA-pFSHβ was independent of increasing the copy number of HSA-pFSHβ gene, which is an important influencing factor on protein production (Supplementary Figure 4).

It is well-known that the Mitogen-Activated Protein Kinase (MAPK) slt2/Mpk1 pathway and Yps1p are required for cell survival under heat stress, osmotic stress, or genotoxic stress (Truman et al., 2007; Bermejo et al., 2008; Soriano-Carot et al., 2012; Gonzalez-Rubio et al., 2021). On the one hand, Slt2/Mpk1 pathway was mutually regulated with the *YPS1* gene, with Slt2 regulating *YPS1* gene expression and *YPS1* gene disruption capable of elevating phosphorylation of Slt2 (Krysan et al., 2005; Gagnon-Arsenault et al., 2006; Miyazaki et al., 2011; Bairwa et al., 2014). Moreover, MAPK signaling pathway, ergosterol synthesis pathway, and the peroxisome pathway were upregulated in robust mutant of produced lipase strain, compared with wild-type strain, indicating that MAPK signaling pathway were involved in recombinant protein production (Lin et al., 2021). Our results were consistent with previous studies that *YPS1* gene disruption enhances Slt2 phosphorylation, thereby reducing HSA-pFSHβ degradation. On the other hand, the production of lipase could be enhanced by overexpressing genes related to antioxidant defence system through reducing the cellular ROS level during methanol-induced fermentation (Lin et al., 2021). Our results showed a similar phenomenon in that the inhibitory effect of *YPS1* gene disruption on HSA-pFSHβ degradation is associated with a decrease in intracellular ROS levels, suggesting that redox homeostasis plays an important role in reducing protein degradation and increasing protein production.

The beneficial effect of *N*-acetyl-L-cysteine (NAC) on HSA-pFSHβ production also implies the importance of maintaining redox homeostasis. As a synthetic precursor of glutathione and an antioxidant, NAC could also improve the intact HSA-pFSHβ production in culture medium through increasing intracellular GSH content in *P. pastoris* (Luo et al., 2021). Moreover, NAC supplementation and *YPS1* gene disruption have synergistic effects on reducing intracellular and extracellular degradation and enhancing yield of recombinant HSA-pFSHβ. This is a good case of improving the production of readily degradable proteins by disrupting *YPS1* gene and supplementing NAC. In addition, the proportion of vacuoles to cells was significantly reduced, indicated that NAC either reduces the size of vacuole or increase the volume of the cytoplasm. It has been reported that the vacuole is the main site of intracellular proteolysis, which is a target of Yps1p within erg6 and pep4 deletion mutant (Sievi et al., 2001). Thus, vacuole plays an important role in degrading recombinant HSA-pFSHβ protein.

It has been reported that *YPT7* gene mutation could reduce the size of the vacuole and regulate the vacuole’s activity in yeast or *in vitro* (Heo et al., 2021). Preventing the nascent protein from targeting vacuole by Ypt7p disruption is an effective method to enhance the secretion of recombinant protein (Marsalek et al., 2019). However, our results indicated that *YPT7* gene disruption decreases intracellular and extracellular intact HSA-pFSHβ production. Unlike NAC, the *YPT7* gene mutation disrupts the vacuolar morphology rather than reducing vacuolar proportion. Moreover, *YPT7* gene mutation inhibits the process of α factor signal peptide by Kex2p, as recombinant HSA-pFSHβ protein that including α factor signal peptide was recognized by FSHβ antibody in cells of F-Δ*ypt7* and Δ*yps1*-Δ*ypt7* strains. Moreover, *YPT7* gene disruption affects the processing of other signaling peptides, including HSA and MSP (Supplementary Figure 5). Thus, we speculated that *YPT7* gene disruption affects the process of signal peptide by disrupting vacuolar function. It has been reported that blocking Kex2p recycling reduced pro-α-factor processing, as the secretion of mature α-factor was reduced (Peterson and Emr, 2001; Bonangelino et al., 2002). Ypt7p is involved in the regulation of transport steps from late endosomes to the vacuole (Schimmoller and Riezman, 1993), and the former plays an important role in the localization of circulating proteins in yeast (Valdivia et al., 2002). Thus, we speculated that Ypt7p participate in the process of α-factor signal peptide sequence through late endosomes.

Ammonium chloride (NH_4_Cl) could reduce the size of vacuole and the degradation of recombinant HSA in yeast, which was related to neutralizing the pH in the Golgi lumen (Kang et al., 2000). However, we found that 0.4M NH_4_Cl was detrimental to HSA-pFSHβ production. We had excluded the effect of pH on HSA-pFSHβ production (Santos et al., 2012). The pH value in culture medium supplemented with NH_4_Cl did not significantly change after 72 h induction (NH_4_Cl for pH 6.92 and control for pH 7.06). Moreover, our previous study has shown that the change of pH value in the culture medium had no obvious effect on HSA-pFSHβ production (Luo et al., 2018). In short, shrinking or disrupting the morphology of vacuole by *YPT7* gene disruption or NH_4_Cl treatment affects the production of recombinant HSA-pFSHβ protein, and the beneficial effect of NAC on HSA-pFSHβ yield does not related to reducing the size of vacuole.

In conclusion, our study confirmed that Yps1p protease mainly cleaved recombinant protein in the culture medium when it was secretory expressed in *P. pastoris*. Disruption of the *YPS1* gene leads to about 30% higher extracellular intact HSA-pFSHβ protein production and about 50% lower degraded fragments yield. Upon combining *YPS1* mutant and NAC supplementation, synergistic effects up to nearly fourfold higher HSA-pFSHβ production and threefold lower HSA-pFSHβ degradation. The mechanism of *YPS1* mutant on HSA-pFSHβ production or degradation is related to the MAPK pathway and maintenance of redox homeostasis. Moreover, our work confirmed that reducing the vacuole site by *YPT7* mutant or NH_4_Cl supplementation affects recombinant HSA-pFSHβ production and Ypt7p participates in signal peptide processing. Our results thus present a versatile method to reduce the decrease of recombinant protein and enhance its production by combining *YPS1* mutants and NAC supplementation.

## Data availability statement

The original contributions presented in this study are included in the article/Supplementary material, further inquiries can be directed to the corresponding author.

## Author contributions

GL, ML, and JY conceived and designed research. GL, ZG, YX, and BK conducted experiments. GL, JL, and ML participated in the discussion and guided the experiment. All authors reviewed and approved the final version of the manuscript.

## Footnotes

1
http://chopchop.cbu.uib.no/

2
http://imagej.nih.gov/ij/
