# Supplementary material for: Enhancement of HSA-pFSHβ production by disrupting YPS1 and supplementing N-acetyl-L-cysteine in Pichia pastoris
Source: Front Microbiol. 2022 Dec 21;13:998647. doi: 10.3389/fmicb.2022.998647 (PMC9810807; doi:10.3389/fmicb.2022.998647)
Supplement: Supplementary file 1 [file Data_Sheet_1.docx]

Supplementary Material

**
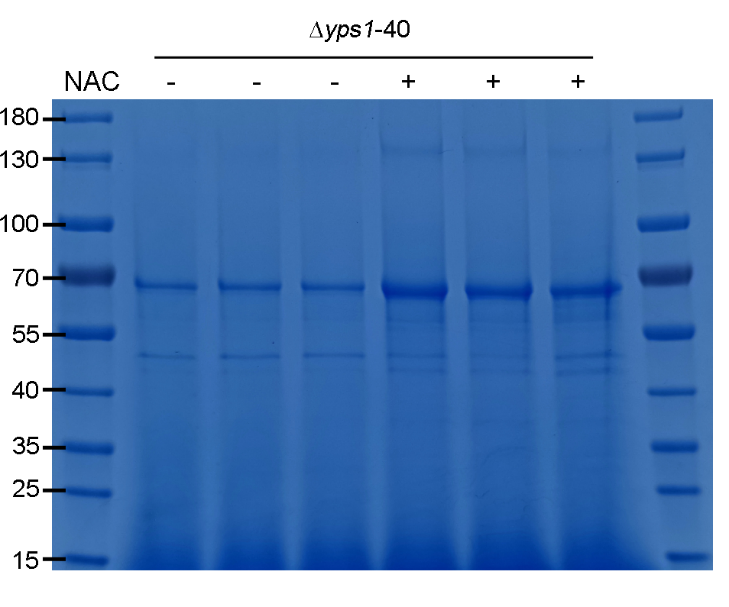
Supplementary Figures**

**

Figure S1.** **SDS-PAGE analysis of HSA-pFSHβ protein in culture medium of *Δyps1-40* supplemented with or without NAC.** +: represent culture medium supplementing with 5 mM NAC, otherwise marked -.

**Figure S2. Determination of alcohol oxidase (AOX) activity in cells of F and *Δyps1* strains.** The cells of F or *Δyps1* strains collected after 72h induction were lysed by using glass beads. The enzyme extracts were obtained by 2860 × g for 10 min at 4°C, and then incubated with the reactive buffer (10 U/mL horseradish peroxidase, 1 mM 4-aminoantipyrine, 4.3 M phenol, and 200 mM methanol in 10 mM PBS (pH7.5)), for 10 min at 37 °C. The OD_500_ of the reactive product was evaluated using an Epoch2 microplate reader (Bio-Tek Instruments). One unit of AOX activity was defined as the amount of enzyme required to produce 1 mmol hydrogen peroxide per minute.





**Figure S3. The cell growth curve (OD_600_) of *Δyps1* and *Δyps1-Δypt7* strains supplemented with 5 mM NAC.** The same amount of cells were inoculated into the induction medium. A linear regression of OD_600_ values against time was performed.





**Figure S4 Relative copy number of HSA-pFSHβ gene in F and *Δyps 1* strains.** The copy number of *Δyps 1* strain was normalized to the copy number of F strain.


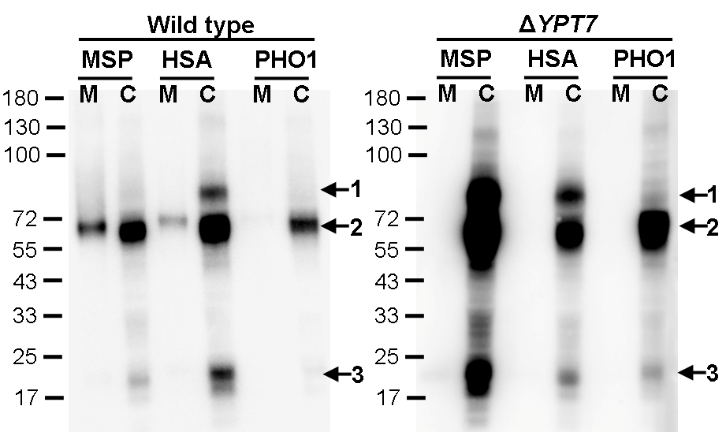


**Figure S5. Western blot analysis of HSA-pFSHβ protein in cells of *YPT7* gene disruption strains.** Blots were probed with antibody specific for human FSHβ monoclonal antibody. Arrow 1: not fully processed HSA-pFSHβ containing signal peptide sequence, arrow 2: HSA-pFSHβ, arrow 3:pFSHβ, M: medium, C: cytosolic protein.

**Table S1 Cell biomass (OD_600_) of different strains at different induction times.**

| **Time** | **F strain** | **Δ*ypt* *7*** | **Δ*yps 1*** | **Δ*yps 1*-Δ*ypt 7*** |
| --- | --- | --- | --- | --- |
| **12 h** | 78.45±0.68 | 80.01±0.61 | 79.11±0.62 | 80.37±0.47 |
| **24 h** | 80.96±1.18 | 84.52±1.01 | 82.00±0.47 | 85.60±0.59 |
| **36 h** | 87.12±0.87 | 90.20±0.73 | 87.68±0.29 | 90.24±0.93 |
| **48 h** | 86.04±0.97 | 91.32±1.37 | 91.00±2.44 | 90.52±0.31 |
| **60 h** | 91.76±1.25 | 94.40±0.90 | 90.88±0.38 | 94.88±0.53 |
| **72 h** | 97.28±1.64 | 95.88±0.69 | 92.88±0.74 | 97.08±1.58 |
| **84 h** | 97.20±1.77 | 98.56±0.70 | 97.24±1.38 | 101.56±0.37 |
| **96 h** | 102.00±0.88 | 106.44±1.69 | 101.84±1.17 | 103.40±0.91 |

Note: The numbers marked in blue indicated that the biomass of Δ*ypt 7* strain was significantly higher than that of F strain, and the numbers marked in red indicate that the cell biomass of Δ*yps 1*-Δ*ypt 7* strain was significantly higher than that of Δ*yps 1* strain. The Student t test was used to determine the statistical significant (p<0.05).
